# Comparison of various classification techniques for supervision of milk processing

**DOI:** 10.1002/elsc.202100098

**Published:** 2021-11-19

**Authors:** Pegah Sadeghi Vasafi, Bernd Hitzmann

**Affiliations:** ^1^ Process Analytics and Cereal Science University of Hohenheim Stuttgart Germany

**Keywords:** anomaly detection, classification methods, milk processing, Raman spectroscopy

## Abstract

Detecting the types of anomalies that can occur throughout the milk processing process is an important task since it can assist providers in maintaining control over the process. The Raman spectrometer was used in conjunction with several classification approaches—linear discriminant analysis, decision tree, support vector machine, and k nearest neighbor—to establish a viable method for detecting different types of anomalies that may occur during the process—temperature and fat variation and added water or cleaning solution. Milk with 5% fat measured at 10°C was used as the reference milk for this study. Added water, cleaning solution, milk with various fat contents and different temperatures were used to detect abnormal conditions. While decision trees and linear discriminant analysis were unable to accurately categorize the various type of anomalies, the k nearest neighbor and support vector machine provided promising results. The accuracy of the support vector machine test set and the k nearest neighbor test set were 81.4% and 84.8%, respectively. As a result, it is reasonable to conclude that both algorithms are capable of appropriately classifying the various groups of samples. It can assist milk industries in determining what is wrong during milk processing.

**ABBREVIATIONS**
kNN: k nearest neighbor
SVM: support vector machine
UHT: ultra‐high temperature processing milk

## 1 INTRODUCTION

Milk and dairy products are well‐known for their benefits to human health. Milk is one of the main components of the human diet and a universal source of nutrients for protein, lactose, vitamins, minerals, and fats [1]. To minimize the production problems, large resource investments are required [2]. Using fast spectroscopy to detect product defects online is beneficial to dairy producers as it can readjust product characteristics or redirect product flow during the production process. During the processing of milk, some abnormal changes—fat and temperature variation, added water and cleaning solution—can happen which threaten the quality and safety of final products. As the fat content of milk is usually set and they are classified by their amount of fat, fat concentration should be consistent and correct during production. Also, the pilot plant is cleansed with cleaning solution and water after production and if some part of the chemical stuff remains in the production line, it creates numerous safety and quality concerns. Moreover, the temperature is the most important option which has to be controlled, as milk is a heat‐treated product. As a result, controlling these changes is a beneficial task [2, 3, 4]. Therefore, controlling the process online is a vital sector that helps a company avoid suffering. In this case, not only detecting the abnormal changes would be an advantage but also it is very important to understand what exactly happened in the processing steps. Therefore, a predictive tool based on online measurement data is needed to monitor every stage of production [3].

Raman spectroscopy has great potential in such applications due to its quick and easy measurement. It has great potential in food quantification and has been applied to food science, especially for dairy technology. A fast‐screening approach for detecting melamine in milk powder with laser Raman spectrometry was developed by Cheng et al. [5] which a detection limit of 0.13% and a good partial least squares (PLS) analysis model were obtained. McGoverin et al. [6] represented the efficiency of Raman spectroscopy at quantifying the protein and fat within skim and whole milk powders. Also, it was effective in the identification of additives such as calcium carbonate. Taking barista foam as an example, the applicability of Raman spectroscopy as a product application parameter index was studied. In order to evaluate the applicability of a purely online system, principal component analysis was used to evaluate the advantages of Raman spectroscopy [07].

Machine learning includes the use of mathematics, statistics, and calculation methods, with the goal of finding effective and accurate classification algorithms. Machine learning algorithms for classification have been successfully used in many different applications, such as food science. The classification problem learning step usually starts with a set of labeled examples containing a training set and a test set. Ciosek et al. [8] classified milk with the use of support vector machine networks. The numerical results of the recognition of milk made differently and with variable fat content have proven to be quite good. A research was done to investigate the use of the least‐squares support vector machine (LS‐SVM) as an alternative multivariate calibration method for the simultaneous quantification of some common adulterants (starch, whey or sucrose) found in powdered milk samples, using near‐infrared spectroscopy with direct measurements by diffuse reflectance and showed promising results [9]. For the automated microbiological quality evaluation of pasteurized vanilla cream, the performance of Fourier transform infrared (FTIR) spectroscopy with support vector machine analysis, was evaluated by Lianou et al. [10]. In the other study, Raman spectral data of milk samples of different species were used for multi‐class classification using a dimensionality reduction technique in combination with a random forest (RF) classifier. With an average accuracy of about 93.7%, precision of 94%, specificity of 97%, and sensitivity of 93%, the suggested technique indicated a considerable potential for a distinction between milk samples of different species [11]. De Lima et al. [12] presented a rapid method for discrimination between lactose and lactose‐free UHT milks using NIRS combined with multivariate classification methods. Among the classification models developed, LDA (linear discriminant analysis) models were more parsimonious due to the use of fewer variables. Although, k nearest neighbor (kNN) classification model was developed to classify control from adulterated milk samples and adulterated milk samples based on the level of adulteration. The results illustrated quite satisfactory predictability, with sensitivity and specificity ranging from 0.66 to 1 [13]. A decision tree (DT) model was utilized to detect post‐calving diseases based on rumination, activity, milk yield, BW and voluntary visits to the milking robot. The overall accuracy of the model was 78%, with a specificity of 87% and a sensitivity of 69%, suggesting its practical value [14]. Using Raman spectroscopy Vasafi et al. [15] could demonstrate that Gaussian process regression as well as autoencoder were able to distinguish between reference milk and manipulated milk, but could not identify which manipulation took place.

**PRACTICAL APPLICATION**

Detection of anomalies during the processing of food would be helpful; however, it is even more important to determine the type of anomalies that happened during the process. It can help companies not only to understand the existence of anomalies in the process but also help them to find the type of it. As a result, classification can assist industries to detect the type of anomalies that happened during milk processing as fast as possible and avoid being suffered.

The main goal of this research was to develop a suitable technique based on data obtained by Raman spectrometer, not only for detecting various changes that can happen during the processing of milk—changes in fat, temperature, added water or contamination of cleaning solution—but also to label which change happened. In this contribution, various classification methods—linear discriminant analysis, decision tree, support vector machine, k nearest neighbor—were tested to find the best method of detection.

## 2 MATERIALS AND METHODS

### 2.1 Sample preparation

In this contribution, ultra‐high temperature processing milk (UHT) with two different fat content of 1.5% and 3.5% were utilized from the brand of “Weihenstephan”, Germany. Samples were kept at the temperature of 5°C before opening the packages. The 1.5% fat milk was utilized as a reference sample. A sample with a concentration of 1.6% fat was prepared by mixing 5 mL of 3.5% fat milk and 95 mL of 1.5% fat milk. In addition, kinds of milk with 1.7% and 1.8% fat content were created by the same procedure. One milliliter of a cleaning solution named “Anti‐Germ” clean A‐N 30″ was diluted with 99 of mL water in order to prepare a common cleaning solution. Therefore, different concentrations of water and cleaning solution (0.05 and 0.1 L/L) were added to 1.5% fat milk. All the samples were measured at 10°C. Finally, 1.5% fat milk was measured after heating up to 15°C and 20°C. The purpose behind this work was to find a proper procedure that can clarify what kind of changes happened during the milk processing. Table 1 presents how various modified samples were created.

**TABLE 1:** Preparation of the abnormal samples

Sample	Preparation method
1.6% fat	Mixing 5 mL of 3.5% fat milk and 95 mL of 1.5% fat milk
1.7% fat	Mixing 10 mL of 3.5% fat milk and 90 mL of 1.5% fat milk
1.8% fat	Mixing 15 mL of 3.5% fat milk and 85 mL of 1.5% fat milk
5% cleaning solution	Adding 5 mL of common cleaning solution to 95 mL of reference milk
5% water	Adding 5 mL of water to 95 mL of reference milk
10% cleaning solution	Adding 10 mL of common cleaning solution to 90 mL of reference milk
10% water	Adding 10 mL of water to 90 mL of reference milk
15°C	Reference samples were measured after heating up to 15°C
20°C	Reference samples were measured after heating up to 20°C

### 2.2 Raman spectroscopy

In this study, an Inno‐Spec Raman 785 spectrometer (Inno‐Spec GmbH, Germany) with a laser excitation wavelength of 785 nm was used to measure the samples. All Raman spectra included scanning with a resolution of 1 cm^–1^ in the spectral range of 65‐3290 cm^–1^. The integration time (IT) used was 20 s. A high‐quality quartz flow cell with a channel length of 1 mm was used for the measurement. To keep the measurement temperature stable at 10°C, the quartz flow cell was connected to the milk source in a cold‐water bath. The flow cell had a capacity of less than 1 mL and was placed 12 mm away from the laser. For each sample, 70% of data were used as the training set while 30% were employed for the test set. One hundred fifty spectra were used for the reference sample and for each modified sample on average 20 spectra were used.

### 2.3 Pre‐processing

Preprocessing has been deemed essential for subsequent data mining tasks and has been determined to be an indispensable part of spectral data analysis. In fact, it has been shown that classification and quantitative models developed based on pre‐processed data generally perform better than models based on raw data. Pre‐processing includes outlier rejection, normalization, filtering, detrending, conversion, folding, and feature selection. The purposes behind spectral preprocessing are better spectral interpretability, greater robustness, and higher precision of post‐classification or quantitative analysis [16]. Therefore, to improve the results, the following preprocessing steps were completed and tested: baseline correction, normalization, multiplicative scatter correction and derivative. Finally, before calculating the standard normal variable (SNV), a Savitzky Golay filter with a second‐order polynomial and a window size of 15 was used to smooth the spectrum. SNV belongs to a group of scatter correction preprocessing methods and can reduce physical variability between samples [17].

### 2.4 Classification algorithms

#### 2.4.1 Overview

Given an unlabeled sample, the classification problem involves determining which class it belongs to, based on a training data set with known class variables. For showing the results of classification a confusion matrix was used. A confusion matrix is an n×n table that summarizes how successful a classification model's predictions were; that is, the correlation between the class and the model's classification. One axis of a confusion matrix is the class that the model predicted, and the other axis is the actual class. n represents the number of different classes. Four concepts were introduced called true positives, true negatives, false positives, and false negatives. A true positive is an outcome where the model correctly predicts the reference samples class. Similarly, a true negative is an outcome where the model correctly predicts the modified samples class. In addition, a false positive is an outcome where the model incorrectly predicts the reference class and a false negative is an outcome where the model incorrectly predicts the modified class. In order to evaluate the classification model, its accuracy was calculated. Classification accuracy is the ratio of correct predictions to total predictions made. In the case of unbalanced data sets, the precision of the classification alone is not the best indicator to evaluate the classifier. Various other performance indicators can be used to gain a more complete understanding of the function of the classifier. The confusion matrix contains enough information to calculate various performance indicators—precision, specificity, and recall. Recall or sensitivity is the metric that measures the accuracy on the positive instances, it can be calculated as true positive/(true positive + false negative). Specificity measures the accuracy on the negative instances and can be defined as true negative/(true negative + false positive). Precision is another metric which is the ratio of true positives to the total of the true positives and false positives [18].

#### 2.4.2 Linear discriminant analysis

LDA was first proposed by Fisher in [19]; today, it is still a complete statistical‐based pattern classification method. Discriminant function analysis is a dimensionality reduction technique commonly used for supervised classification problems. It is used to model differences in groups, such as separating two or more classes. It can be used to project features in space from high‐dimensional to low‐dimensional ones. Therefore, it focuses on the separation ability among the classes. In this technique, a new axis is created based on maximizing the distance between the means of each category and minimizing the variation within each category [20]. For the training set, each class was named by a number and the test set was predicted by using the function of predict in MATLAB with the full covariance function.

#### 2.4.3 Decision tree

By its simplest description, decision tree analysis is a divide and conquer approach for classification. Decision trees can be used to discover features and extract patterns in large databases that are important for discrimination and predictive modelling [21]. A decision tree consists of nodes at which a variable is tested. A variable can be a nominal or numerical value and in the latter case, the test usually determines whether the variable's value is greater or less than a predetermined constant, resulting in a two‐way split. A variable is selected to split the data set at the first node (root node). For each possible test outcome at the node, a branch is made ending in a daughter node. The process can be repeated recursively for each branch, using only those records that actually reach the branch. If at any time all records at a node have the same classification, that part of the tree stops developing [18]. The same procedure as for other techniques was done and the accuracy of the model was calculated.

#### 2.4.4 Support vector machine

Support vector machine (SVM) is a supervised learning algorithm that is well suited for determining patterns in complex data sets. It performs the classification by finding a hyperplane that maximizes the margin between classes. The vector determines the hyperplane is considered the support vector. The algorithm performs the classification and learns from the examples to predict the classification of never‐before‐seen data [22]. To do so, two inputs are needed: training data set and test data set. The class label file clarifies each training example, in this case, each set of samples is represented by a specific number. The goal of model selection is to adjust the hyperparameters (penalty parameters and any kernel parameters) of the SVM classification to achieve the lowest test error, such as the lowest probability of misclassification from unseen test examples [23]. As predictor variables the intensity values depending on the wavelength were utilized, as the response values, each group of samples were put equal to a specific number. The cubic function was implemented as the kernel function. The classification learner was used for the model calculation, for prediction the function predict was applied. Binary classification is employed for classification tasks with two classes while multi‐class classification is implemented for classification tasks with more than two classes. Heuristic methods can be used to split a multi‐class classification problem into multiple binary classification datasets and train a binary classification model each. One‐vs‐All is a heuristic method for using binary classification algorithms for multi‐class classification whereas the One‐vs‐One strategy splits a multi‐class classification into one binary classification problem per each pair of classes. It involves splitting the multi‐class dataset into multiple binary classification problems. In this case, all the strategies were tested to find the best algorithms and finally, One‐vs‐All was utilized.

#### 2.4.5 K nearest neighbor

The k nearest neighbor is one of the simplest machine learning algorithms, based on the fact that objects close to each other will show similar characteristics. Therefore, if the characteristics of a sample are obvious, it is easy to predict the characteristics of its neighbors. k is a positive small integer which indicates how many neighbors are considered. The k nearest neighbors are selected based on distance metric and here, the Euclidean was employed [23]. In this contribution, for the training set, each class was named by a number and k was equal to 3. In MATLAB, the classification learner was used for the model calculation, for prediction the function predict was applied.

## 3 RESULTS AND DISCUSSION

### 3.1 Linear discriminant analysis

**FIGURE 1:**
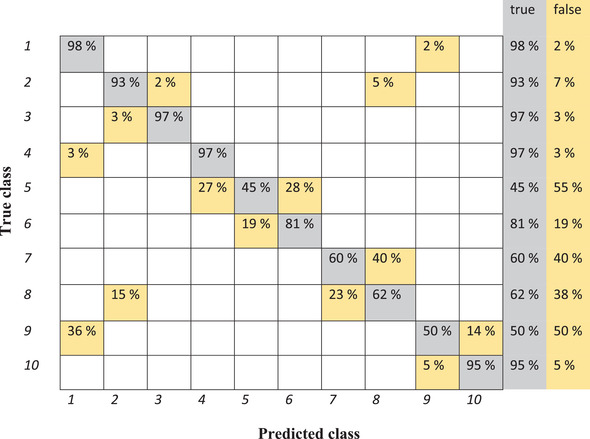
Confusion matrix of classification of various samples from training set by means of linear discriminant analysis. 1: Reference sample, 2: 10% water, 3: 5% water, 4: 1.6% fat, 5: 1.7% fat, 6: 1.8% fat, 7: 5% cleaning solution, 8: 10% cleaning solution, 9: 15°C, 10: 20°C (misclassifications are shown in yellow)

Linear discriminant analysis shows an accuracy of 89.8% for the training set and 67.8% for the test set. Figure 1 represents the result of using linear discriminant analysis for classification in a confusion matrix.

As can be seen in Figure 1, 98% of the reference samples are classified correctly in group one. However, 2% of the mentioned samples are classified in the group of samples which were measured at 15°C (false negative). In contrast, 3% of the samples with 1.6% fat and 36% of samples measured at 15°C are wrongly categorized as reference samples (false positive). The highest wrong classification is related to sample number 7 which is named 5% cleaning solution in which 40% of spectra are classified as the samples with 10% cleaning solution. Although, while 45% of samples with 1.7% fat are correctly classified, 27% of this sample classified as 1.6% fat and 28% as 1.8% fat. By a closer look at Figure 1, just 62% of the sample with 10% cleaning solution classified correctly. The mentioned sample is classified into wrong groups, 23% in the group of 5% cleaning solution and 15% in the group of 10% water. As the training model did not work well, the test set is not discussed here.

### 3.2 Decision tree

**FIGURE 2:**
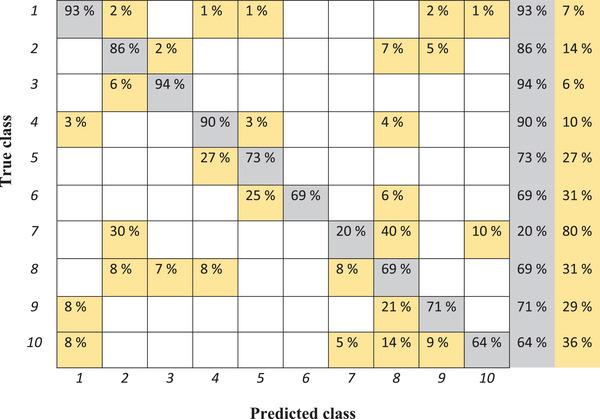
Confusion matrix of classification of various samples from training set by means of decision tree. 1: Reference sample, 2: 10% water, 3: 5% water, 4: 1.6% fat, 5: 1.7% fat, 6: 1.8% fat, 7: 5% cleaning solution, 8: 10% cleaning solution, 9: 15°C, 10: 20°C (misclassifications are shown in yellow)

The decision tree shows the accuracy of 83.9% for the training set and 61% for the test set. The confusion matrix obtained by using the decision tree and the training set for classification is presented in Figure 2.

According to Figure 2, there are a lot of misclassifications where each sample is classified into several irrelevant groups. Reference samples are classified as 10% water, 1.6 and 1.7% fat and samples measured at 15°C and 20°C. The best classification is referred to sample with 5% water, of which 94% are classified truly and 6% are categorized as 10% water. The worst classified sample is referred to the sample with 5% cleaning solution, while just 20% of spectra are classified correctly. Forty percent of mentioned samples are in the group of 10% cleaning solution, 30% in the group of 10% water, and 10% in the group of samples measured at 20°C.

### 3.3 Support vector machine

**FIGURE 3:**
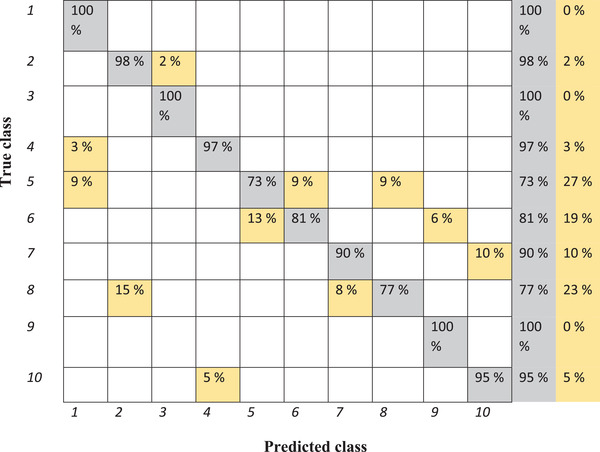
Confusion matrix of classification of various samples from training set by means of support vector machine. 1: Reference sample, 2: 10% water, 3: 5% water, 4: 1.6% fat, 5: 1.7% fat, 6: 1.8% fat, 7: 5% cleaning solution, 8: 10% cleaning solution, 9: 15°C, 10: 20°C (misclassifications are shown in yellow)

Figure 3 demonstrates the result of using a support vector machine to classify the training set in the confusion matrix.

The accuracy of the model is 96% for the training set and 81.4% for the test set. According to Figure 3, all the reference samples are classified correctly in group number one. While all the samples with 5% water are classified correctly, 2% of samples with 10% water are classified as 5% water. Three percent of the sample with 1.6% fat and 9% of samples with 1.7% fat are classified wrongly as reference sample.

As shown in Table 2, while most of the sample represents the high value for recall, the sample with 10% water shows a value of just 11%. By accurate investigation, it was found that this sample is mainly classified as 5% water and minimally as 10% cleaning solution. The value of recall for the sample with 10% cleaning solution is 67% when some of the samples are classified as 10% water. The calculated specificities of samples are quite high, while the lowest one is equal to 93% for milks measured at 20°C. The computed precision values for samples with various fat content are 100% implies the ability of this method for correct categorizing of fat content. The lowest precision values are 50% and 55% for the samples with 10% water and measured at 20°C, respectively.

**TABLE 2:** Calculated recall, specificity, and precision of the test set for each sample using support vector machine

Sample	Recall (sensitivity)	Specificity	Precision
Reference sample	100%	98%	93%
10% water	11%	98%	50%
5% water	100%	94%	67%
1.6% fat	80%	96%	67%
1.7% fat	100%	100%	100%
1.8% fat	80%	100%	100%
10% cleaning solution	67%	100%	100%
5% cleaning solution	75%	100%	100%
15°C	100%	100%	100%
20°C	100%	93%	55%

### 3.4 K nearest neighbor

**FIGURE 4:**
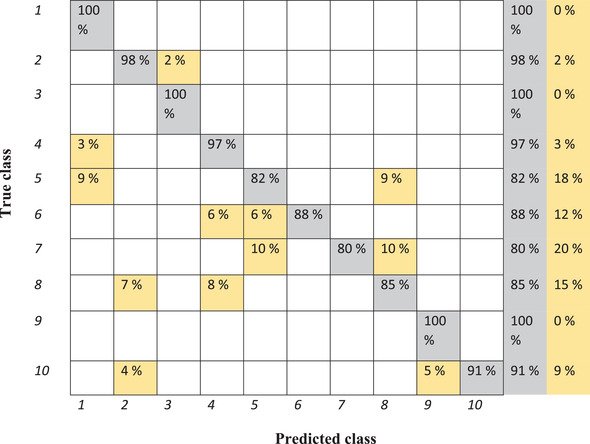
Confusion matrix of classification of various samples from training set by means of k nearest neighbor. 1: Reference sample, 2: 10% water, 3: 5% water, 4: 1.6% fat, 5: 1.7% fat, 6: 1.8% fat, 7: 5% cleaning solution, 8: 10% cleaning solution, 9: 15°C, 10: 20°C (misclassifications are shown in yellow)

The result of classification of the training set by using k nearest neighbor in the confusion matrix is presented in Figure 4.

The accuracy of the model is 96.4% for the training set and 84.8% for the test set. According to Figure 4, all the reference samples are classified correctly; however, 3% of 1.6% fat and 9% of 1.7% fat are categorized as the reference samples (the same as support vector machine). In comparison with other samples, 5% cleaning solution is not classified as good as others. Ten percent of the mentioned sample is classified wrongly as 1.7% fat and 10% as the samples with 10% cleaning solution. Six percent of the sample with 1.8% fat is categorized wrongly as 1.7% fat and also 6% as 1.6% fat. Samples measured at 20°C are classified quite good; however, 4% and 5% of the spectra are classified as 10% water or 15°C, respectively. Low numbers of spectra related to 10% cleaning solution are classified as 10% water and 1.6% fat wrongly.

As can be seen in Table 3, recall values for the reference sample, 5% water and cleaning solution, 1.7% fat, and 20°C are equal to 100%. Although, the lowest value is contributed to the 10% water which is mostly classified as the 5% water wrongly. The recall value for 10% cleaning solution is 67%, where this sample is mostly categorized as 10% water. In addition, the mentioned value for samples with 1.8% fat content is equal to 75%. The calculated specificity of samples is quite high, while the lowest value is 92% contributed to 5% water in the milk. Despite a hundred per cent precision for most of the samples, samples with 5 and 10% water show the precision of 62% and 60%, respectively. The precision value for samples measured at 20°C is 83% and for the reference sample is equal to 93%.

**TABLE 3:** Calculated recall, specificity, and precision of test set for each sample using k nearest neighbor

Sample	Recall (sensitivity)	Specificity	Precision
Reference sample	100%	98%	93%
10% water	56%	94%	62%
5% water	100%	92%	60%
1.6% fat	80%	100%	100%
1.7% fat	100%	100%	100%
1.8% fat	75%	100%	100%
10% cleaning solution	67%	100%	100%
5% cleaning solution	100%	100%	100%
15°C	80%	100%	100%
20°C	100%	98%	83%

## 4 CONCLUDING REMARKS

The use of a process analyzer to monitor milk processing helps suppliers to maintain product quality and safety before filling and packing. In this research, Raman spectroscopy was used for developing an innovative approach that can detect anomalies from reference processing in a by‐pass to reduce the probability of quality‐defect recalls. To determine the best machine learning methodology for classifying various anomalies, a variety of classification methods—linear discriminant analysis, decision tree, support vector machine, and k nearest neighbors—were used. The results demonstrate that decision tree and linear discriminant analysis models, with the accuracy of 61% and 67.8% for the test set, respectively, are unable to correctly predict the classes. Also, by taking a look at the confusion matrix of the training set, it is clear that these two methods classify the reference samples as the modified samples and vice versa. Consequently, continuing with them was not fruitful in this application. In contrast, the support vector machine and k nearest neighbor, perform well in the categorization of diverse groups, with the accuracy of 81.4% and 84.8% for the test set, respectively. In this case, the most important thing is that anomalies can be separated from the reference signals. Classifying the various anomalies would be good but not essential. Therefore, it would be necessary to distinguish samples with abnormal changes from the reference sample which support vector machine and k nearest neighbor did. Both procedures show high values of recall, specificity, and accuracy for the reference sample (100%, 98%, and 93%, respectively), indicating that these methods are capable of classification. In addition, these values are quite high for the modified samples imply the fact that most of the abnormal signals are classified correctly by these methods. Therefore, these two methods might work as well for the classification of other spectroscopic applications.

In general, it can be stated that support vector machine and k nearest neighbor are capable of accurately detecting and identifying various anomalies during milk processing, allowing the milk industry to respond quickly to the situation.

## CONFLICT OF INTEREST

The authors have declared no conflict of interest.

## DATA AVAILIBILTY STATEMENT

The data that support the findings of this study are available from the corresponding author upon reasonable request.
